# P-1123. Characterization of microbiological isolates in the newborn unit of the San Ignacio university hospital between 2018 and 2022

**DOI:** 10.1093/ofid/ofae631.1310

**Published:** 2025-01-29

**Authors:** Monica Maria Perez, Ana Maria Bertolotto, Adriana Del Pilar Montealegre, Juan Carlos Lopez, Yaris Anzully Vargas, Maria Alejandra Suarez, Ingrid Mayerly Gomez, Gloria Cecilia Cortes

**Affiliations:** Pontificia Universidad Javeriana, Bogotá D.C., Distrito Capital de Bogota, Colombia; Hospital Universitario San Ignacio, Bogota, Distrito Capital de Bogota, Colombia; Hospital Universitario San Ignacio, Bogota, Distrito Capital de Bogota, Colombia; Hospital Universitario San Ignacio, Bogota, Distrito Capital de Bogota, Colombia; Hospital Universitario San Ignacio, Bogota, Distrito Capital de Bogota, Colombia; Hospital Universitario San Ignacio, Bogota, Distrito Capital de Bogota, Colombia; Hospital Universitario San Ignacio, Bogota, Distrito Capital de Bogota, Colombia; Hospital Universitario San Ignacio, Bogota, Distrito Capital de Bogota, Colombia

## Abstract

**Background:**

Identifying local microbial profiles and antibiotic sensitivities is an important part of antimicrobial stewardship programs in order to prevent overuse of antibiotics. The purpose of this study was to characterize the microbiological isolates from patients hospitalized in the newborn care unit of the Hospital Universitario San Ignacio from January 1, 2018 to December 31, 2022.

Total number of microorganisms isolated

**Figure:**
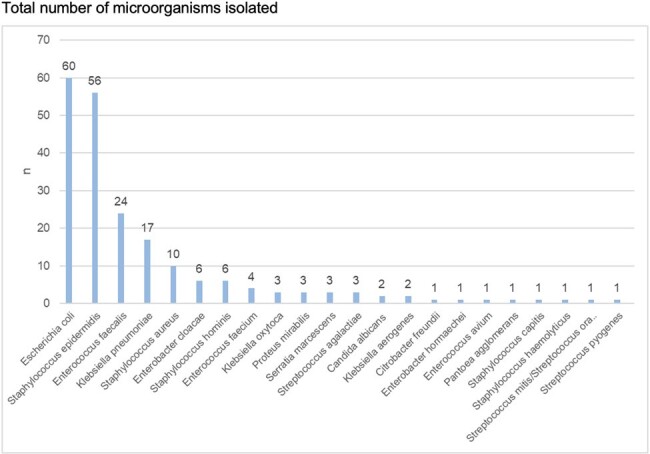

1

**Methods:**

An observational, descriptive, historical single cohort study was conducted. Positive cultures of sterile samples (blood, urine, spinal fluid) from patients hospitalized. Demographic, clinical, and microbiological data of patients with positive cultures were also collected.

**Figure:**
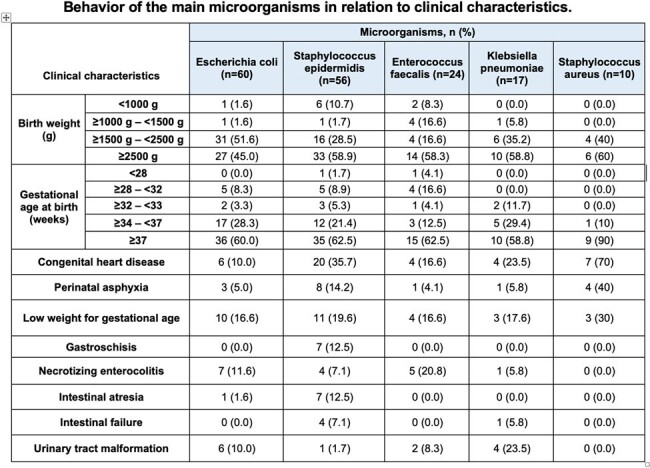

**Results:**

A total of 207 positive cultures of sterile samples were identified (97 blood, 108 urines, 2 spinal fluid). The most frequent pathogens isolated were *E. coli* 28.9%, *S. epidermidis* 27%, *Enterococcus faecalis* 11.5%, *Klebsiella pneumoniae* 8.2% and *Staphylococcus aureus* 4.8%. *E. coli* and *S. epidermidis* being the main microorganisms isolated 28% each were the main pathogens associated with neonatal sepsis (NS). NS was predominantly late-onset >72 hours 94.6%. Early-onset sepsis was less common 5.3% and was associated with *E. coli* 5.4% and *S. agalactiae* 18.1%.

Healthcare-associated infections (34.7%), and catheter-associated bloodstream infections (CLABSI) were the most common. Congenital heart disease (*P* = .000) was the main comorbidity related to CLABSI, as well as gastroschisis (*P* = 0.002) and intestinal atresia (*P* = 0.033)

Multidrug-resistant (MDR) microorganisms were isolated in 12 instances (5.8%). Extended Spectrum Beta-Lactamase (ESBL) was 50%. Congenital heart disease was the only comorbidity with a statistically significant relationship with infection by MDR microorganisms (*P* = 0.026).

Clinical and sociodemographic characteristics of the multidrug-resistant microorganism population.

**Figure:**
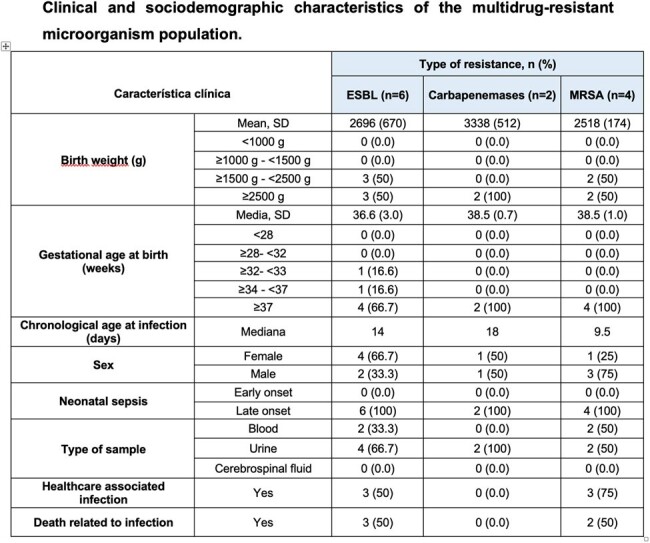

**Conclusion:**

This is the first study in our environment to know the basal factors of infections in a population at risk, the patterns of resistance and possible association with the most prevalent pathologies and, based on this, to define the strategies of antimicrobial stewardship.

**Disclosures:**

**All Authors**: No reported disclosures

